# Formatting and gene-based delivery of a human PD-L1 single domain antibody for immune checkpoint blockade

**DOI:** 10.1016/j.omtm.2021.05.017

**Published:** 2021-06-04

**Authors:** Robin Maximilian Awad, Quentin Lecocq, Katty Zeven, Thomas Ertveldt, Lien De Beck, Hannelore Ceuppens, Katrijn Broos, Yannick De Vlaeminck, Cleo Goyvaerts, Magali Verdonck, Geert Raes, Alexander Van Parys, Anje Cauwels, Marleen Keyaerts, Nick Devoogdt, Karine Breckpot

**Affiliations:** 1 Laboratory for Molecular and Cellular Therapy, Department of Biomedical Sciences, Vrije Universiteit Brussel, 1090 Brussels, Belgium; 2 *In Vivo* Cellular and Molecular Imaging Laboratory, Department of Medical Imaging, Vrije Universiteit Brussel, 1090 Brussels, Belgium; 3 Laboratory of Cellular and Molecular Immunology, Department of Bioengineering Sciences, Vrije Universiteit Brussel, 1050 Brussels, Belgium; 4 Myeloid Cell Immunology Lab, VIB Center for Inflammation Research, 1050 Brussels, Belgium; 5 Cytokine Receptor Laboratory, Flanders Institute of Biotechnology, VIB Medical Biotechnology Center, Faculty of Medicine and Health Sciences, Ghent University, 9000 Ghent, Belgium; 6 Nuclear Medicine Department, UZ Brussel, 1090 Brussels, Belgium

**Keywords:** programmed death-ligand 1, single domain antibody, nanobody, gene therapy

## Abstract

Monoclonal antibodies that target the inhibitory immune checkpoint axis consisting of programmed cell death protein 1 (PD-1) and its ligand, PD-L1, have changed the immune-oncology field. We identified K2, an anti-human PD-L1 single-domain antibody fragment, that can enhance T cell activation and tumor cell killing. In this study, the potential of different K2 formats as immune checkpoint blocking medicines was evaluated using a gene-based delivery approach. We showed that 2K2 and 3K2, a bivalent and trivalent K2 format generated using a 12 GS (glycine-serine) linker, were 313- and 135-fold more potent in enhancing T cell receptor (TCR) signaling in PD-1^POS^ cells than was monovalent K2. We further showed that bivalent constructs generated using a 30 GS linker or disulfide bond were 169- and 35-fold less potent in enhancing TCR signaling than was 2K2. 2K2 enhanced tumor cell killing in a 3D melanoma model, albeit to a lesser extent than avelumab. Therefore, an immunoglobulin (Ig)G1 antibody-like fusion protein was generated, referred to as K2-Fc. K2-Fc was significantly better than avelumab in enhancing tumor cell killing in the 3D melanoma model. Overall, this study describes K2-based immune checkpoint medicines, and it highlights the benefit of an IgG1 Fc fusion to K2 that gains bivalency, effector functions, and efficacy.

## Graphical abstract

**Figure:**
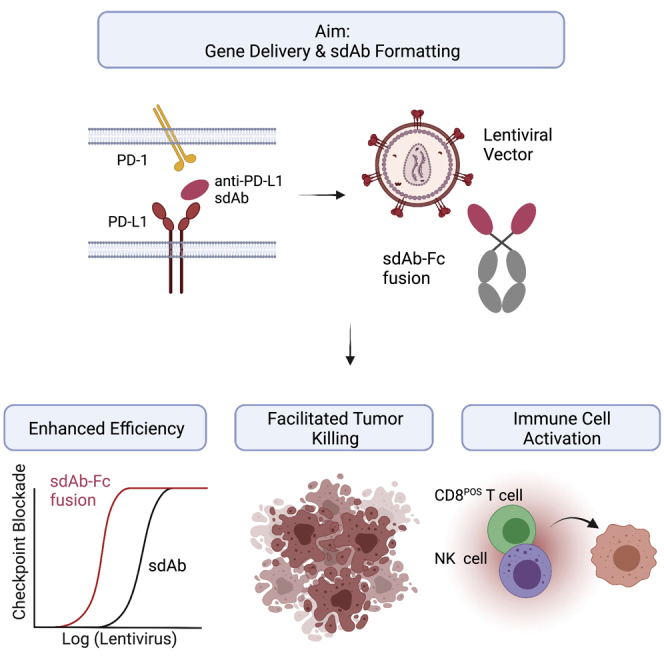

## Teaser

Awad et al. describe how formatting of a human PD-L1 sdAb impacts the efficiency of immune checkpoint blockade upon lentiviral-mediated sdAb delivery. The authors systematically study how an increase in avidity, choice of linkage, and gain of Fc functions affect the sdAb performance when delivered either genetically or in the protein form.

## Introduction

Blockade of inhibitory immune checkpoints using monoclonal antibodies (mAbs) has proven clinical value in a variety of solid tumors and has become a mainstay therapy option.^1^, ^2^, ^3^, ^4^, ^5^, ^6^ Blockade of the inhibitory immune checkpoint consisting of programmed cell death protein 1 (PD-1, CD279) and its ligand PD-L1 (B7-H1, CD274) has received much attention, as this immune checkpoint hampers the antitumor immune response at multiple levels, i.e., the priming and effector phases.^7^^,^^8^

PD-1 is a type 1 transmembrane receptor that is mainly expressed on the surface of T cells upon their activation.^9^ Engaging PD-1 on T cells interferes with T cell receptor (TCR) signaling, thereby preventing excessive stimulation, and maintaining tolerance to self-antigens.^10^ This poses a barrier for antitumor immunity, as PD-L1 is highly expressed on dendritic cells that need to orchestrate the antitumor immune response.^11^ In addition, PD-L1 is often abundantly expressed on immune cells and cancer cells in the tumor microenvironment (TME).^12^, ^13^, ^14^ Expression of PD-L1 on the surface of cancer cells, myeloid cells, and lymphoid immune cells in the TME paralyzes the effector function of PD-1^POS^ T cells. Also, expression of PD-L1 on the surface of T cells within the TME promotes tumor escape through suppressive back signaling and installing a tumor-promoting program in PD-1^POS^ macrophages.^14^ Moreover, the PD-L1 signalosome in cancer cells promotes cancer cell survival through regulation of resistance to pro-apoptotic stimuli, such as interferons, thereby posing another barrier to antitumor immunity.^15^

We previously developed K2, a single-domain antibody fragment (sdAb, also known as a nanobody) with nanomolar affinity for human PD-L1.^16^ sdAbs are small-sized antibody derivatives with high potential in immune-oncology applications.^17^ We showed that K2 competes with the mAb avelumab for binding to PD-L1, allowing head-to-head comparison of the ability of K2 and avelumab to enhance T cell activation by dendritic cells and tumor cell killing by T cells.^16^^,^^18^ Both K2 and avelumab efficiently bound to PD-L1 on the surface of tumor cells and promoted tumor cell killing by activated T cells.^16^ Since sdAbs are rapidly cleared from the circulation, the adaptation for therapeutic purposes often requires engineering of the sdAb format or the exploitation of alternative delivery approaches.

In this study, we explored whether a gene-based delivery method and/or formatting of K2 could circumvent the need for repeated dosing. We addressed the importance of valency, method of sdAb linkage, and the inclusion of an immunoglobulin (Ig)G1 Fc fragment.

## Results

### Restoration of TCR signaling upon interaction of PD-1^POS^ T cells with PD-L1^POS^ tumor cells requires high doses of K2-encoding lentiviral vectors (LVs)

We previously showed using an *in vitro* 3D tumor model that repeated dosing of K2 can support tumor cell killing by activated peripheral blood mononuclear cells (PBMCs).^16^ However, sdAbs are characterized by fast clearance *in vivo*, necessitating a smart approach to ensure sufficiently high K2 levels in the TME upon translation of this approach to an *in vivo* setting. Therefore, we evaluated gene-based delivery of K2 to tumor cells.

**Figure 1:**
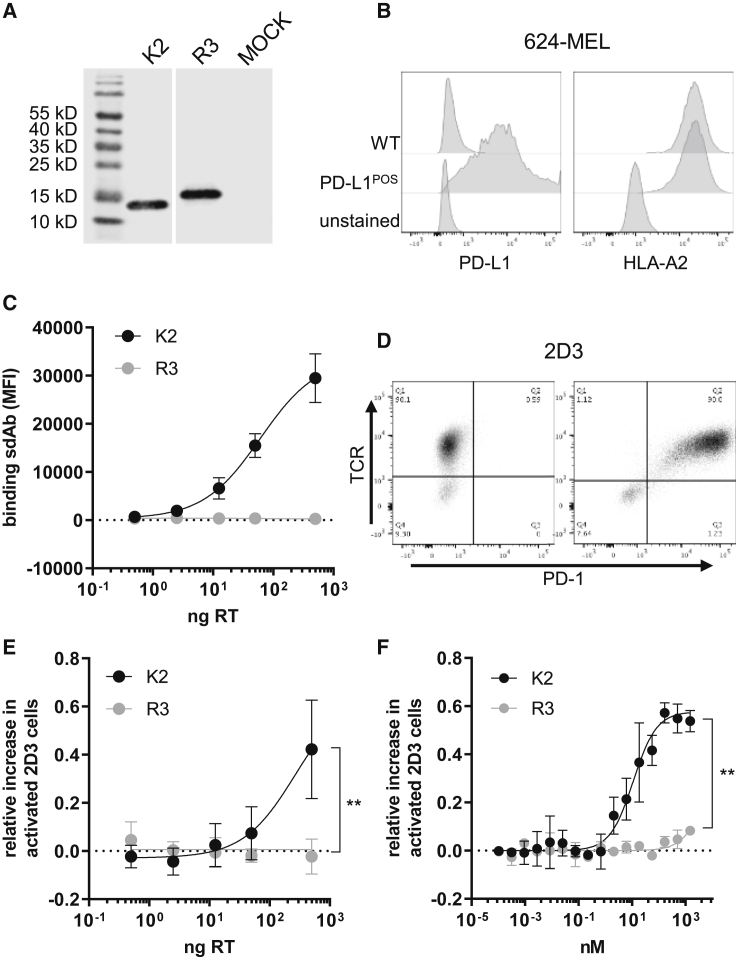
Functional K2 can be expressed by 293T and 624-MEL cells upon lentiviral transduction and restores 2D3 functionality (A) Western blot confirms the production and secretion of His-tagged K2 and control sdAb R3 by transfected 293T cells. One representative experiment is shown (n = 3). (B) Representative histogram showing PD-L1 and HLA-A2 expression on 624-MEL cells that were lentivirally transduced to express high PD-L1 levels (n = 6). (C) Amount of lentivirus (LV), expressed as ng of reverse transcriptase (RT), used to transduce 624-MEL PD-L1^POS^ cells, versus amount of sdAb binding on transduced 624-MEL PD-L1^POS^ cells, determined by extracellular staining and flow cytometry (n = 3). (D) Representative dot plot showing 2D3 cells that were electroporated with mRNA to express the TCR recognizing gp100 in the context of HLA-A2 (left), or both the TCR and PD-1 (right) (n = 9). (E) PD-1^POS^ TCR^POS^ or PD-1^NEG^ TCR^POS^ 2D3 cells were co-cultured with PD-L1^POS^ 624-MEL cells transduced with varying amounts of K2- or R3-encoding LVs. GFP expression of 2D3 cells was determined as a measure for 2D3 cell activation. Relative increase in activated 2D3 cells was calculated as the ratio of PD-1^POS^ 2D3 cells over PD-1^NEG^ 2D3 cells from co-cultures with sdAb-producing 624-MEL cells minus the ratio from co-cultures with untransduced 624-MEL cells. The graph summarizes the relative increase in activated 2D3 cells as mean ± standard deviation (SD) of three independent experiments (n = 3). (F) Activation in TCR signaling in PD-1^POS^ TCR^POS^ 2D3 cells versus PD-1^NEG^ TCR^POS^ 2D3 cells when co-cultured with PD-L1^POS^ 624-MEL cells in the presence of different concentrations of K2 or R3 protein. The graph summarizes the activation in TCR signaling as the mean ± SD of three independent experiments (n = 3). ∗∗p < 0.01.

We cloned the 360-bp genetic code for K2 fused to an N-terminal Igκ secretion signal and a C-terminal histidine (His)-tag into a lentiviral transfer plasmid. The genetic code of sdAb R3, previously referred to as R3B23, binding to the idiotype of 5T2 multiple myeloma cells^19^, which serves as a control sdAb, was cloned in a similar fashion (Figure S1A). To ensure production of the sdAbs, human embryonic kidney 293T cells were transfected with the transfer plasmids, after which the presence of the sdAbs in supernatants was detected by western blot. As shown in Figure 1A, both K2 and R3 were produced and secreted by the 293T cells and exhibited the expected sizes of 13 and 15 kDa. The lentiviral transfer plasmids were used for production of second-generation LVs that were quantified by measuring the amount of reverse transcriptase (RT) (Figure S1B). Doses ranging from 0.5 to 500 ng of RT were used to transduce 624-MEL melanoma cells that express high levels of human leukocyte antigen (HLA)-A2 and that were modified to express PD-L1 (Figure 1B). Production of K2 by these cells was evaluated in flow cytometry by detecting K2 bound to PD-L1 on the cell surface of these PD-L1^POS^ 624-MEL cells. We observed a dose-dependent increase in surface-bound K2 on PD-L1^POS^ 624-MEL cells (Figure 1C). These lentivirally engineered PD-L1^POS^ 624-MEL cells served as antigen-presenting cells in the 2D3 reporter assay, which was previously developed to study PD-1/PD-L1 immune checkpoint blocking medicines.^20^ Herein, TCR-defective Jurkat 2D3 cells are electroporated with mRNA encoding PD-1 and/or mRNA encoding the TCRα/β chain of a TCR that recognizes glycoprotein (gp)100_280–288_ in the context of HLA-A2 (Figure 1D). Activation of TCR signaling in PD-1^NEG^ 2D3 cells results in upregulation of the transcription factor NFAT, which regulates the expression of a green fluorescent protein (GFP). This GFP expression is decreased in PD-1^POS^ 2D3 cells because of the PD-1 interaction with PD-L1 on 624-MEL PD-L1^POS^ cells (Figures S1C and S1D). We showed that PD-L1^POS^ 624-MEL cells that were transduced with high doses of K2-encoding, but not R3-encoding, LVs were able to restore TCR signaling in PD-1^POS^ TCR^POS^ 2D3 cells (Figure 1E). For comparison, we performed a 2D3 assay using PD-L1^POS^ 624-MEL cells and increasing doses of exogenously added K2 or R3 purified sdAbs, which showed maximal restoration of TCR signaling when using 100–1,000 nM K2 (Figure 1F).

### Increasing the valency of K2 reduces the doses of K2-encoding LVs required to restore TCR signaling upon interaction of PD-1^POS^ T cells with PD-L1^POS^ tumor cells

Since high amounts of LVs were necessary to effectively block the PD-1/PD-L1 interaction, we next addressed whether increasing the functional affinity of K2 could reduce the doses of LVs required to achieve maximal TCR signaling in PD-1^POS^ 2D3 cells.

**Figure 2:**
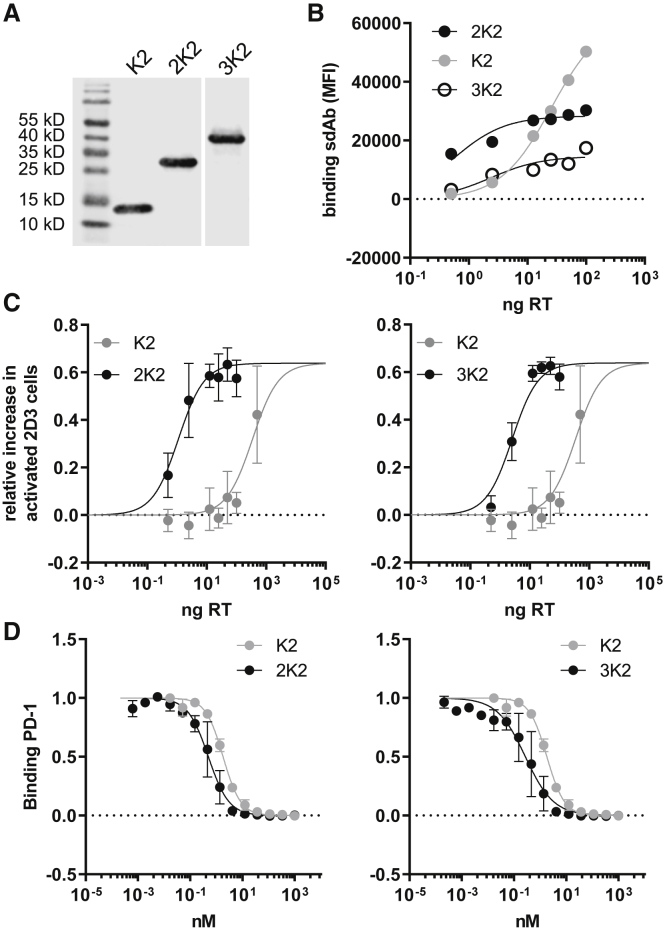
Increasing the valency of K2 reduces the necessity for high doses of LVs (A) Western blot was performed on supernatant derived from 293T cells transfected with lentiviral transfer plasmids encoding His-tagged K2, 2K2, or 3K2. One representative experiment is shown (n = 3). (B) PD-L1^POS^ 624-MEL cells were transduced with different amounts of LVs encoding K2, 2K2, or 3K2. sdAbs on the surface of the transduced cells were stained using anti-His-tag antibodies. The graph shown is representative of three independent experiments (n = 3). (C) Modified PD-L1^POS^ 624-MEL cells were co-cultured with PD-1^POS^ TCR^POS^ or PD-1^NEG^ TCR^POS^ 2D3 cells. The activation of 2D3 cells was calculated as described in the legend to Figure 1 based on GFP expression. The graph summarizes the activation in TCR signaling as mean ± SD (n = 3). (D) 624-MEL PD-L1^POS^ cells were incubated with recombinant human PD-1 and varying concentrations of K2, 2K2, or 3K2. PD-1 on the cell surface was measured using flow cytometry (n = 3).

We designed a bivalent and trivalent K2 in which two or three K2 molecules were coupled using a 12 GS (glycine-serine) linker, referred to as 2K2 and 3K2, respectively. Similar formats were generated for R3, referred to as 2R3 and 3R3 (Figure S2A). We cloned the genetic code for 2K2, 3K2, 2R3, and 3R3 fused to an N-terminal Igκ secretion signal and a C-terminal His-tag into the transfer plasmid. To ensure production of the multivalent sdAbs, human embryonic kidney 293T cells were transfected with the transfer plasmids, after which the presence of the sdAbs in supernatants was detected by western blot. As shown in Figure 2A, the multivalent K2 formats were produced at similar levels as the monovalent K2 by the 293T cells and exhibit the expected sizes of 28 and 41 kDa. The lentiviral transfer plasmids were used for production of second-generation LVs that were characterized by measuring the amount of RT (Figure S2B). Doses ranging from 0.5 to 100 ng of RT were used to transduce PD-L1^POS^ 624-MEL cells. Binding of K2, 2K2, and 3K2 on PD-L1 on the surface of these cells was evaluated by flow cytometry to verify production of the different K2 formats. We showed that all multivalent K2 constructs were able to bind PD-L1, while binding of corresponding R3 constructs was not observed (data not shown). We moreover observed a dose-dependent increase in binding of PD-L1 with distinct differences between K2, 2K2, and 3K2 (Figure 2B). The lentivirally engineered PD-L1^POS^ 624-MEL cells were co-cultured with PD-1^POS^ and TCR^POS^ 2D3 cells, after which expression of GFP was measured in flow cytometry as a measure of TCR signaling. We showed that 2K2 and 3K2 were 313- and 135-fold more potent in enhancing TCR signaling in PD-1^POS^ TCR^POS^ 2D3 cells than monovalent K2 (Figure 2C). To assess the biophysical properties of the sdAb formats more quantitatively, K2, 2K2, and 3K2 were harvested from lentivirally transduced 293T cell supernatants, concentrated, quantified, and first used in a flow cytometry-based competition study with recombinant human PD-1. Both 2K2 and 3K2 showed superior PD-L1 blocking abilities compared with K2 (Figure 2D). We further assessed the binding kinetics of K2, 2K2, and 3K2 using surface plasmon resonance (SPR). We found that 2K2 and 3K2 showed improved binding compared to K2, with a visual clearly improved off rate (Figure S2C).

### Changing the link in bivalent K2 formats impacts the doses of bivalent K2-encoding LVs required to restore TCR signaling upon interaction of PD-1^POS^ T cells with PD-L1^POS^ tumor cells

As 2K2 showed a more favorable outcome than 3K2, we continued with designing various bivalent constructs (Figure S3A). In 2K2, we used a 12 GS linker, which is a flexible linker that consists of stretches of glycine and serine residues. To achieve a better separation of the two K2 molecules, we increased the copy number of these GS stretches to 30. This K2 format is referred to as 2K2-30GS. We moreover generated a bivalent K2 in which two K2 proteins with a carboxyl-terminal IgA-hinge linker and a cysteine residue were post-translationally linked through a disulfide bond.^21^ It has been shown that carboxyl-terminal linkage avoids spatial occupancy, which is sometimes observed with genetically linked sdAbs.^22^ This K2 format is referred to as 2K2-DS.

**Figure 3:**
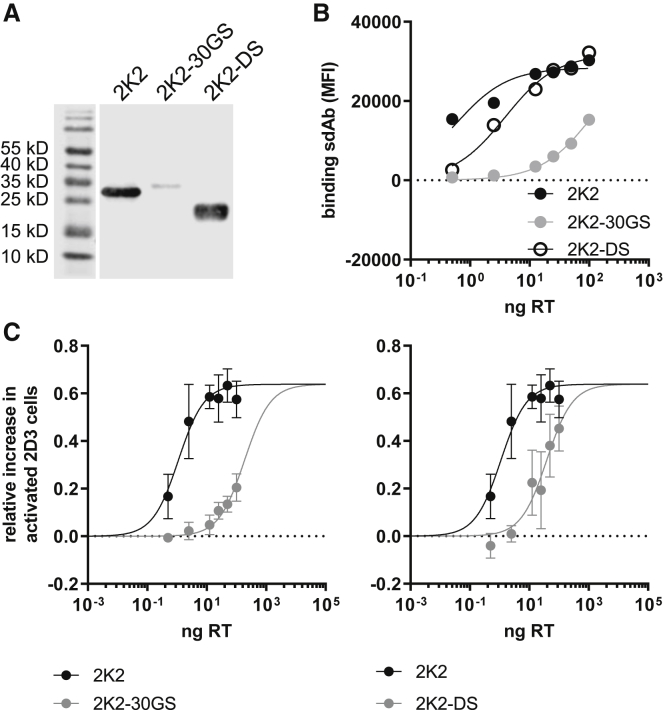
Changing the link in bivalent K2 negatively impacts the efficiency of PD-1/PD-L1 blockade (A) Western blot was performed on supernatant derived from 293T cells transfected with lentiviral transfer plasmids encoding His-tagged 2K2, 2K2-30GS, or 2K2-DS. One representative experiment is shown (n = 3). (B) PD-L1^POS^ 624-MEL cells were transduced with different amounts of LVs encoding 2K2, 2K2-DS, or 2K2-30GS. sdAbs on the surface of the transduced cells were stained using anti-His-tag antibodies. One representative graph is shown (n = 3). (C) Modified PD-L1^POS^ 624-MEL cells were co-cultured with PD-1^POS^ TCR^POS^ or PD-1^NEG^ TCR^POS^ 2D3 cells. The activation of 2D3 cells was calculated as described in the legend to Figure 1 based on GFP expression. The graph summarizes the activation in TCR signaling as mean ± SD (n = 3).

We cloned the genetic code for 2K2-30GS and 2K2-DS and the corresponding R3 formats fused to an N-terminal Igκ secretion signal and a C-terminal His-tag into the transfer plasmid. To ensure production of the multivalent sdAbs, human embryonic kidney 293T cells were transfected with the transfer plasmids, after which the presence of the sdAbs in supernatants was detected by western blot. As shown in Figure 3A, 2K2, 2K2-30GS, and 2K2-DS exhibit the expected sizes (in reducing conditions) of, respectively, 28, 29, and 20 kDa. While 2K2 and 2K2-DS formats were efficiently produced, the 2K2-30GS format lagged behind in yield. Nonetheless, we continued with the production and characterization of second-generation LVs using these lentiviral transfer plasmids (Figure S3B). Doses varying from 0.5 to 100 ng of RT were used to transduce PD-L1^POS^ 624-MEL cells. Binding of 2K2, 2K2-30GS, and 2K2-DS on PD-L1 on the surface of these 624-MEL cells was evaluated in flow cytometry to verify production of the different K2 formats. We showed that the binding profile of 2K2 and 2K2-DS to PD-L1 was comparable, while binding of PD-L1 by 2K2-30GS was low, even when the PD-L1^POS^ 624-MEL cells were transduced with high doses of LVs (Figure 3B). The lentivirally engineered PD-L1^POS^ 624-MEL cells were co-cultured with PD-1^POS^ and TCR^POS^ 2D3 cells to evaluate TCR signaling as a function of PD-1/PD-L1 blockade. We showed that 2K2-30GS and 2K2-DS were 2- and 9-fold more potent in enhancing TCR signaling in PD-1^POS^ TCR^POS^ 2D3 cells than was monovalent K2; however, it did not reach the level of PD-1/PD-L1 blockade that was observed with 2K2 (Figure 3C). We also produced concentrated supernatant from transduced 293T cells containing 2K2-DS and used it in a flow cytometry-based competition study with recombinant human PD-1 protein, and in SPR to assess the binding kinetics toward PD-L1. In neither assay did 2K2-DS show improvements in binding to or blocking PD-L1 compared to K2 (Figure S3C). A comparative assay with 2K2-30GS is lacking, as its expression level is too low to perform this assessment.

### 2K2 is similarly as potent as avelumab in enhancing TCR signaling

**Figure 4:**
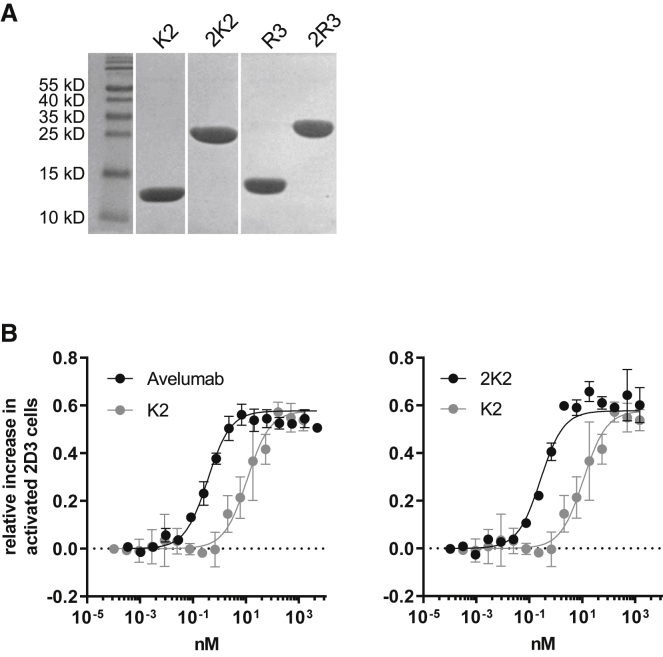
2K2 is similarly as potent as avelumab in enhancing TCR signaling (A) SDS-PAGE of 20 μg of purified K2, 2K2, R3, or 2R3 (n = 1). (B) PD-L1^POS^ 624-MEL cells were co-cultured with PD-1^POS^ TCR^POS^ or PD-1^NEG^ TCR^POS^ 2D3 cells in the presence of serially diluted K2, 2K2, or avelumab. The activation of 2D3 cells was calculated as described in the legend to Figure 1 on the basis of GFP expression. The graph summarizes the activation in TCR signaling as mean ± SD (n = 2).

Of the formats tested, 2K2 showed the best performance in the restoration of TCR signaling when delivered by LVs. To enable a side-by-side comparison with avelumab, a mAb that competes with K2 for binding to PD-L1,^16^ we produced 2K2 in a protein format. The sequence of K2, 2K2, and the corresponding controls R3 and 2R3 were cloned into the pHEN6 plasmid to fuse them to a carboxyl-terminal His-tag. Bacteria were transformed and subsequently sdAbs were isolated from the periplasmatic extract by performing immobilized metal affinity chromatography followed by size-exclusion chromatography. The purity and expected sizes of the sdAbs, i.e., 15 kDa for K2 and R3 and 30 kDa for 2K2 and 2R3, were confirmed by SDS-PAGE (Figure 4A). To evaluate the capacity to enhance TCR signaling, we co-cultured PD-L1^POS^ 624-MEL cells with PD-1^POS^ TCR^POS^ 2D3 cells in presence of different amounts of K2, 2K2, avelumab, or control sdAbs. Bivalency shifts the concentration-response curve of K2 into the picomolar range, comparable to the effects achieved with avelumab. The half-maximal effective concentrations (EC_50_s) of 2K2 and avelumab were 42- and 31-fold lower than the EC_50_ of K2. 2K2 shows a comparable or slightly better potential than avelumab in enhancing TCR signaling (Figure 4B).

### Tumor killing is facilitated upon lentiviral delivery of 2K2 in an allogeneic 3D melanoma model

**Figure 5:**
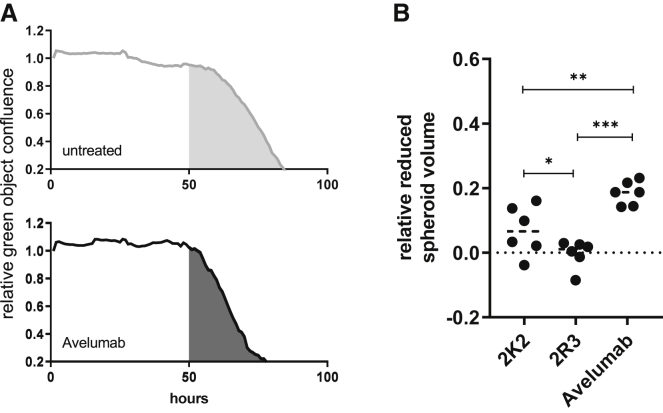
Lentiviral delivery of 2K2 to 3D tumors *in vitro* has therapeutic efficacy (A and B) Activated PBMCs were added to 624-MEL GFP^POS^ cells that were grown in 3D tumors. 3D tumors were transduced with LVs encoding 2K2 or 2R3 or were treated with avelumab. 3D tumors that were not treated served as a control. The green object confluence, representing viable tumor cells, was measured every hour for 5 consecutive days using the IncuCyte Zoom live cell imaging system. The area under the curve (AUC) of treated 3D tumors was normalized to the AUC of non-treated 3D tumors, after which treatment modalities were compared. (A) Representative decline in green fluorescent confluence in a non-treated (gray tinted) and avelumab treated (black) condition. (B) Relative reduced tumor volume calculated as 1 – AUC_TREATED_/AUC_MOCK_ (n = 6). ∗p < 0.05, ∗∗p < 0.01, ∗∗∗p < 0.001.

We demonstrated that 2K2 can effectively activate TCR signaling. Next, we investigated the effect of 2K2 on tumor cell killing. To ensure that the sdAbs or avelumab did not directly affect proliferation of 624-MEL PD-L1^POS^ cells, we evaluated cell growth in the presence of 2K2, 2R3, or avelumab. We could not observe differences in 624-MEL proliferation (Figure S4A). We generated 3D tumor cultures from wild-type 624-MEL cells. We transduced these 3D tumors with LVs encoding 2K2 or 2R3 to mimic a relevant *in vivo* setting where LVs are administered intratumorally. The 3D tumors were co-cultured with PBMCs from HLA-A2^POS^ donors in the presence of interleukin (IL)-15 to stimulate immune cell-mediated tumor rejection. Untransduced 624-MEL 3D tumors and 3D tumors that were not treated or treated with avelumab served as negative or positive controls, respectively. We demonstrated that 2K2 can facilitate 3D tumor destruction when delivered by LVs. However, the effects achieved with 2K2 were less pronounced than the effects achieved with avelumab (Figure 5B). We evaluated composition of immune cells and their activation during the killing assay using flow cytometry (Figure S4B). Avelumab administration led to an increase in CD8^POS^ T cells and a decrease in CD4^POS^ T cells and natural killer (NK) cells. On NK cells, avelumab led to increased CD134 and CD137 expression. Moreover, we observed a strong decline in CD16 expression when avelumab was administered. While avelumab effectively modulated immune cell composition and activation, 2K2 did not cause significant changes (Figure S4C).

### Tumor cell killing is achieved in an allogeneic 3D melanoma model upon lentiviral delivery of an IgG1 antibody-like K2 format

**Figure 6:**
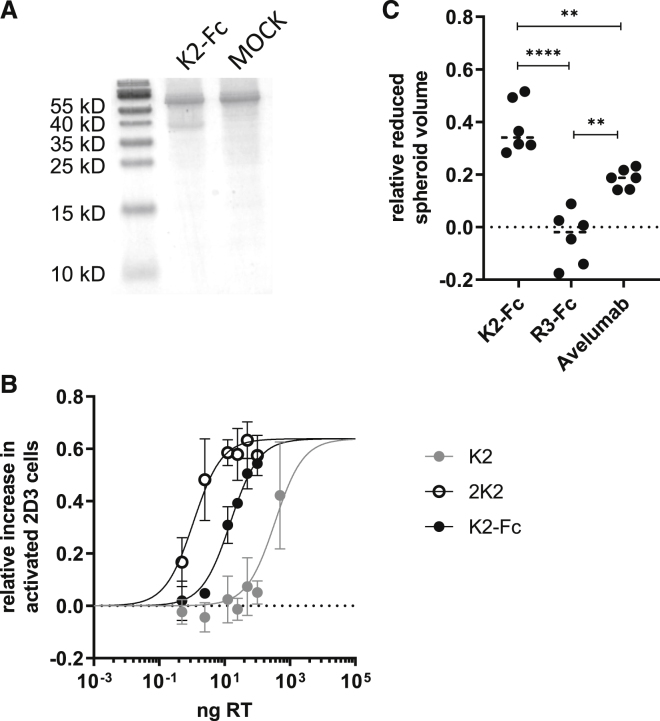
Lentiviral delivery of K2-Fc enhances tumor cell killing (A) SDS-PAGE was performed on supernatants derived from 293T cells transduced with lentiviral vectors encoding K2-Fc (n = 3). (B) PD-L1^POS^ 624-MEL cells were transduced with varying amounts K2-, 2K2-, or K2-Fc-encoding LVs and subsequently co-cultured with PD-1^POS^ TCR^POS^ or PD-1^NEG^ TCR^POS^ 2D3 cells. The activation of 2D3 cells was calculated as described in the legend to Figure 1 on the basis of GFP expression. The graph summarizes the activation in TCR signaling as mean ± SD (n = 2). (C) Activated PBMCs were added to 624-MEL GFP^POS^ cells that were grown in 3D tumors. 3D tumors were transduced with LVs encoding K2-Fc or R3-Fc or were treated with avelumab or left untreated. The green object confluence, representing viable tumor cells, was measured every hour for 5 consecutive days using the IncuCyte Zoom live cell imaging system. The AUC of treated 3D tumors was normalized to the AUC of non-treated 3D tumors. Treatment modalities were compared as relative reduced tumor volume (n = 6). ∗∗p < 0.01, ∗∗∗∗p < 0.0001.

**Figure 7:**
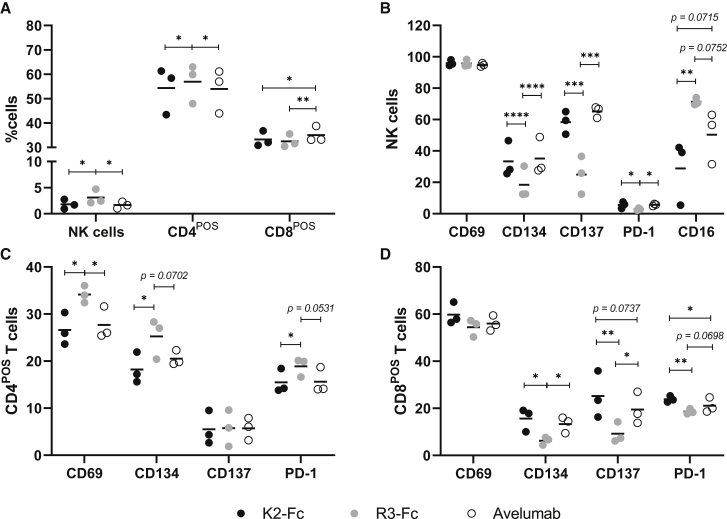
K2-Fc effectively modulates immune cell composition and activation (A–D) Activated PBMCs were added to 624-MEL cells that were grown in *in vitro* 3D tumors. 3D tumors were transduced with LVs encoding K2-Fc or R3-Fc or were treated with avelumab. Once spheroid size was visibly reduced, immune cells were isolated and analyzed using flow cytometry (n = 3). ∗p < 0.05, ∗∗p < 0.01, ∗∗∗p < 0.001, ∗∗∗∗p < 0.0001.

The previous experiment showed that 2K2 facilitates tumor killing in a 3D melanoma model and that avelumab facilitated tumor killing more efficiently than did 2K2. IL-15 is an activator for a variety of immune cells, including, among others, T cells and NK cells. Especially the latter will exhibit cytolytic activity after encountering IL-15. One of these mechanisms is antibody-dependent cell-mediated cytotoxicity (ADCC) when CD16 on the surface of NK cells binds to the Fc tail of an IgG1 antibody. Avelumab contains an IgG1 domain. Since 2K2 was shown to be more efficient in restoring TCR signaling than avelumab, effector functions mediated by the IgG1 domain of avelumab could be responsible for the more efficient tumor rejection. To evaluate whether IgG1 and ADCC are responsible for the enhanced tumor cell killing, we genetically fused the sequences of K2 via a hinge region to the sequence of the Fc region of IgG1 comprising an E333A substitution that increases the capacity to induce ADCC.^23^ We cloned this construct, referred to as K2-Fc, into a lentiviral transfer plasmid. In analogy, a corresponding control construct with the sequence of sdAb R3 was created (Figure S5A). The lentiviral transfer plasmids were used for the production of second-generation LVs that were characterized by measuring the amount of RT (Figure S5B). We transduced 293T cells to produce K2-Fc. The sdAb-containing supernatants were concentrated and the presence of K2-Fc was confirmed using SDS-PAGE (Figure 6A). The supernatants were used in a flow cytometry-based competition study with recombinant human PD-1. Analogous to avelumab and compared to K2, K2-Fc showed superior PD-1 blocking capacities (Figure S5C). We further assessed the binding kinetics of K2-Fc using SPR. We found that K2-Fc has a similar affinity as avelumab for binding to PD-L1 (Figure S5D). To evaluate the capacity of LV-mediated delivery of K2-Fc to block the PD-1/PD-L1 interaction, we transduced 624-MEL PD-L1^POS^ cells with doses ranging from 0.5 to 100 ng of RT of K2-Fc- or R3-Fc-encoding LVs. The cells were co-cultured with PD-1^POS^ and TCR^POS^ 2D3 cells, after which GFP expression was measured by flow cytometry as a measure of TCR signaling. We demonstrated that K2-Fc is 23-fold more and 14-fold less potent in enhancing TCR signaling in PD-1^POS^ TCR^POS^ 2D3 cells than K2 and 2K2, respectively (Figure 6B). To determine whether K2-Fc affects the viability of 624-MEL PD-L1^POS^ cells, we evaluated cell growth in the presence of K2-Fc and R3-Fc. K2-Fc did not directly affect 624-MEL PD-L1^POS^ proliferation (Figure S5E). Next, we created 3D tumors from wild-type 624-MEL cells and transduced these 3D tumors with LVs encoding K2-Fc and R3-Fc. After transduction, 3D tumors were co-cultured with PBMCs that were stimulated with IL-15. The decrease in 3D tumor confluence was monitored as a measure for tumor cell killing. Increased tumor cell killing was observed in 3D tumors transduced with LVs encoding K2-Fc compared to 3D tumors transduced with LVs encoding R3-Fc or 3D tumors treated with avelumab (Figure 6C). We evaluated the immune cell composition and activation. Analogous to avelumab, K2-Fc led to a decrease of CD4^POS^ T cells and NK cells (Figure 7A). Within the NK cell fraction and equally to avelumab, K2-Fc elevated CD134, CD137, and PD-1 expression. K2-Fc strongly abrogated CD16 (Figure 7B). Within the CD4^POS^ T cells, K2-Fc led to a decrease in CD69, CD134, and PD-1 expression (Figure 7C). In the CD8^POS^ T cell fraction K2-Fc led to upregulation of CD134, CD137, and PD-1 (Figure 7D).

## Discussion

A challenge in implementation of the immune checkpoint blockade for cancer therapy is to limit the activation of the immune system to the site of interest, the tumor, as systemic immune activation withholds the risk of severe adverse effects, such as autoimmunity and nonspecific inflammation.^24^^,^^25^ Therefore, local delivery of antibodies targeting regulatory immune checkpoints, including PD-1/PD-L1, has been studied with delivery strategies ranging from peritumoral injection of mAbs to their gene-based delivery using viral and nonviral vectors.^26^, ^27^, ^28^ The genetic delivery of therapeutic antibodies might offer a number of advantages as compared to their intratumoral/peritumoral administration, of which omitting the need for repeated injection and as such reducing the therapy cost is one.^29^ Several viral delivery systems have been studied in the context of immune checkpoint inhibition among other adeno-associated viral vectors and retroviral vectors.^30^ In this study, we chose LVs as a delivery system for human PD-L1 targeting sdAbs, as LVs were previously shown to efficiently deliver their cargo to mainly tumor cells upon intratumoral delivery,^31^ and because LVs have been used in a clinical setting for therapy for primary immunodeficiencies and cancers.^32^ To mimic delivery of LVs to tumors, we used 3D tumors and activated PBMCs to which LVs are delivered. This *in vitro* model allows evaluation of the therapeutic efficacy of the delivered cargo,^16^ enabling us to demonstrate efficient PD-1/PD-L1 blockade after the administration of LVs encoding 2K2 or K2-Fc to these 3D tumors.

Prior to these 3D tumor models, we investigated the therapeutic efficacy of monovalent K2 in the 2D3 reporter assay that was previously developed to evaluate the efficacy of PD-L1 blocking agents.^20^ We showed that high doses of LVs were required to enable full activation of PD-1^POS^ 2D3 cells upon interaction with modified PD-L1^POS^ 624-MEL cells. Since such high doses of LVs are prohibitive for *in vivo* translation, we evaluated whether formatting of K2 could result in similar therapy efficacy, but using lower doses of LVs. First, we increased the valency, as this strategy has been previously used to enhance the efficacy of sdAbs in cancer therapy.^33^ We evaluated 3K2, a trivalent, and 2K2, a bivalent format of K2 and observed that the number of LVs encoding 2K2 that was needed to restore TCR signaling in PD-1^POS^ 2D3 cells upon interaction with PD-L1^POS^ 624-MEL cells was about 2- and 313-fold less than the number of LVs encoding 3K2 and K2, respectively. Contrary to our observations in this functional assay, 3K2 shows superior blocking abilities compared to 2K2 when delivered as protein. This suggests that the higher efficiency of 2K2-encoding LVs could be attributed to better transduction efficiencies. The choice of an appropriate linker between sdAbs is an important consideration in rational sdAb drug design and has been shown to be a decisive factor influencing the biological activity and expression.^33^, ^34^, ^35^, ^36^ We showed that bivalent constructs generated using a 30 GS linker or disulfide bond, as opposed to a 12 GS linker, were 35- and 169-fold less potent in enhancing TCR signaling. This could in part be explained by the lower expression levels of 2K2-30GS and highlights that potency screening of sdAb formats with different linkers when pursuing a genetic approach should take into consideration factors such as transcription, translation, post-translational modification, and secretion of the encoded sdAb. Therefore, the optimal sdAb linker for gene delivery has to be empirically determined based on the sdAbs and the target antigen. Next, to genetic linkage between the carboxyl and amino terminus of K2, we evaluated the bivalent construct 2K2-DS, which is post-translationally assembled via disulfide bonds. It has been shown earlier that the functionality of sdAbs could be impaired due to spatial occupancy of the linker in a genetically fused sdAb and that sdAbs linked via carboxyl termini retain their biological activity.^22^^,^^37^ However, we could not observe improvements of this construct when delivered by LVs. Conversely, we observed that the blocking efficiency was not increased and that the affinity profile regarding dissociation rates did not show improvements compared to monovalent K2. It has been shown earlier that next to spontaneous dimerization, glutathione capping can occur, leading to the formation of monovalent entities.^21^

Avelumab has a high affinity compared to other clinically approved PD-L1 antibodies.^38^ We found that K2 has a lower affinity for PD-L1, and it is therefore less potent in restoring TCR signaling. Bivalency could increase the functional affinity and potency to a level comparable to avelumab. However, when we compared lentivirally delivered 2K2 to exogenously added avelumab in the 3D tumor model, we observed that avelumab was more potent in stimulating tumor cell killing by IL-15-activated PBMCs. Therefore, we decided to link K2 to a human IgG1 Fc fragment through which K2 gained bivalency, effector functions, and efficacy, as shown in the 3D tumor model in which lentiviral delivery of K2-Fc stimulated tumor cell killing more efficiently than did avelumab. However, avelumab contains a native Fc domain whereas K2 was fused to a modified IgG1 Fc domain with an enhanced affinity for the FcγRIII and increased capacity to induce ADCC.^23^^,^^39^ It is likely that this modified Fc domain enhanced tumor cell killing more effectively than did the native Fc domain of avelumab. Both avelumab and K2-Fc effectively modulated immune cell composition and activation in a similar way. Chimeric antibody constructs have been generated with sdAbs that compete with durvalumab for binding to PD-L1. This construct, referred to as KN035 or envafolimab, has been used in a subcutaneous therapy strategy and was shown to have low immunogenicity, a good tumor penetrance, and antitumor efficacy in preclinical models.^38^ Envafolimab was administered subcutaneously as well in a phase I clinical trial for treatment of patients with solid tumors, showing safety and evidence of antitumor activity.^40^ These results have pushed the evaluation of envafolimab in a phase II study for microsatellite instability-high advanced solid tumors and a phase III trial for patients with cholangiocarcinoma in China. The results obtained so far with subcutaneous administration of envafolimab together with the data presented in this study on lentiviral delivery of K2-Fc warrant further *in vivo* research into the use of K2-Fc as an immune checkpoint medicine.

## Materials and methods

### Reagents

Mouse anti-His-tag antibodies (clone AD1.1.10, Bio-Rad, Belgium) were used as primary antibodies for the staining of His-tagged sdAbs in flow cytometry and western blot. As a secondary antibody for detection of His-tagged sdAbs on PD-L1^POS^ 624-MEL cells in flow cytometry, phycoerythrin (PE)-conjugated anti-mouse IgG antibody (clone A85-1, BD Biosciences, Belgium) was used. As a secondary antibody for the detection of His-tagged sdAbs on western blot membranes, near infrared IRDye 800CW anti-mouse IgG antibody was used (LI-COR Biosciences, USA). Brilliant Violet (BV)421-conjugated anti-human IgG-Fc antibody (HP6017, BioLegend, USA) was used to detect the binding of recombinant human PD-1-Fc to PD-L1^POS^ 624-MEL cells in flow cytometry. PD-L1 expression was confirmed with PE-CF594-conjugated anti-PD-L1 antibodies (MIH1, BD Biosciences). HLA-A2 expression on 624-MEL cells was verified using fluorescein isothiocyanate (FITC)-conjugated anti-HLA-A2 antibodies (BB7.2, BD Biosciences) or BV421 conjugated anti-HLA-A2 antibodies (BB7.2, BioLegend). 2D3 cells were characterized using allophycocyanin (APC)-H7-conjugated anti-CD8 antibodies (clone SK1, BD Biosciences), BV421-conjugated anti-PD-1 antibodies (EH12.2H7, BioLegend), and PE-conjugated anti-TCRα/β antibodies (clone IP26, BioLegend). To increase the specificity of immunofluorescent staining, FcR blocking reagent (Miltenyi Biotec, Bergisch Gladbach, Germany) was used. Antibodies used for the characterization of immune cells were Alexa Fluor 700-conjugated anti-CD45 (HI30, BioLegend), BV605-conjugated anti-CD3 (SK7, BD Biosciences), PE/cyanine 7-conjugated anti-CD4 (SK3, BioLegend), BV421-conjugated anti-CD8 (RPA-T8, BioLegend), PE-Dazzle594-conjugated anti-CD56 (5.1H11, BioLegend), peridinin chlorophyll protein/cyanine 5.5-conjugated anti-CD69 (FN50, BioLegend), APC-conjugated anti-CD134 (Ber-ACT35, BioLegend), PE-conjugated anti-CD137 (4B4-1, BioLegend), FITC-conjugated anti-PD-1 (A17188B, BioLegend), APC-Fire750-conjugated anti-CD16 (3G8, BioLegend), and Zombie Aqua (BioLegend) were used to discriminate between live and dead cell populations. Human gp100_280–288_ peptide (Eurogentec, Belgium) was used for pulsing 624-MEL cells in the 2D3 assay. Avelumab (Bavencio, provided by Merck [EMD Serono] and Pfizer) was used in the 2D3 assay and the 3D melanoma model. Biacore consumables were from Cytiva (Machelen, Belgium). Recombinant His-tagged human PD-L1 protein (R&D Systems, Minneapolis, MN, USA) was used for SPR analysis of PD-L1 binding sdAbs. Recombinant human PD-1-Fc and biotinylated recombinant human PD-1 were used for flow cytometry-based competition studies.

### Cell lines

293T cells were acquired from the American Type Culture Collection (ATCC, USA) and cultured in Dulbecco’s modified Eagle’s medium (DMEM) (Sigma-Aldrich, Belgium) supplemented with 10% fetal bovine serum (FBS) (Harlan, the Netherlands), 2 mM l-glutamine, 100 U/mL penicillin, and 100 μg/mL streptomycin (Sigma-Aldrich). 2D3 cells were generated as described earlier^20^ and cultured in Iscove’s modified Dulbecco’s medium (IMDM) (Thermo Scientific) supplemented with 10% FBS, 2 mM l-glutamine, 100 U/mL penicillin, and 100 μg/mL streptomycin. HLA-A∗0201^POS^ 624-MEL cells were provided by S.L. Topalian (National Cancer Institute, Baltimore, MD, USA) and were cultured in Roswell Park Memorial Institute (RPMI) 1640 medium (Sigma-Aldrich) supplemented with 10% fetal clone I serum (Thermo Scientific), 2 mM l-glutamine, 100 U/mL penicillin, 100 μg/mL streptomycin, 1 mM sodium pyruvate, and nonessential amino acids (Sigma-Aldrich).

PBMCs obtained from HLA-A∗0201^POS^ donors were provided by the service of Hematology of the Brussels University Hospital. These cells were collected using a blood cell separator (Spectra Optia apheresis system, Terumo BCT, USA) by processing up to 7 L of whole blood. The PBMC concentrate was subjected to elutriation using a semi-automatic counter-flow elutriation instrument (Elutra cell separation system, version 1.1, Terumo BCT) using a user defined profile. The program that was used collects cells in five fractions. The chamber rotation speed was maintained at 2,400 revolutions per minute for fractions 1 through 4, and the media flow rate was maintained at 37 mL per minute for fraction 1, 97.5 mL per minute for fraction 2, 103.4mL per minute for fraction 3, and 103.9 mL per minute for fractions 4 and 5. Fraction 5 consists of the cells remaining in the separation chamber and that are collected with the rotor turned off. Fractions 2–4 were pooled and further referred to as PBMCs. This study was approved by the Ethical Committee of the UZ Brussel (2013/198).

### LV production and characterization

The packaging plasmid pCMVΔR8.9 and the envelope plasmid pMD.G were a kind gift from D. Trono (University of Geneva, Italy). The pHR′ transfer plasmids encoding PD-L1 and GFP were previously described.^7^^,^^41^ DNA sequences encoding the different sdAb formats containing a murine Igκ secretion signal and a His-tag were designed *in silico* using SnapGene in order to generate the transfer plasmids encoding the different sdAb formats. The codons of the sequences were optimized using the codon optimization tool of Integrated DNA Technologies (IDT) and synthesized by IDT (Belgium). DNA fragments were foreseen with overhangs allowing the ligation into the pHR′ vector digested with the restriction enzymes EcoRI and BamHI. DNA fragments were ligated using the NEBuilder HiFi DNA assembly master mix (New England Biolabs [NEB], USA) according to the manufacturer’s instructions. The plasmids were transformed in NEB 5-alpha competent *Escherichia coli* bacteria (NEB) and subsequently in XL1Blue *E. coli* using the TransformAid bacterial transformation kit (Thermo Scientific). The selection of clones was performed on ampicillin (0.1 μg/mL, Bristol-Myers Squib, Belgium)-enriched Luria-Bertani (LB) agar plates. Large bacterial cultures in LB medium containing 0.1 μg/mL ampicillin were used to extract plasmid DNA (Maxiprep kit, QIAGEN, Germany). The plasmid DNA yield was determined by spectrophotometry.

The production of LVs and their characterization using the colorimetric RT assay (Roche, Germany) was performed as detailed elsewhere.^42^^,^^43^

### Transduction

624-MEL cells were seeded at 10^5^ cells per well in a six-well tissue culture plate (Thermo Scientific) and treated overnight with 800 μL of DMEM containing 100 ng of RT of LVs. Cells were expanded and subsequently sorted using a BD FACSAria cell sorter (BD Biosciences) on GFP^HIGH^ cells. PD-L1^POS^ 624-MEL cells were generated as described earlier.^16^

### 2D3 assay

PD-L1^POS^ 624-MEL cells were seeded at 5 × 10^4^ cells per well in a six-well plate and left for 24 h to adhere. These adherent cells were transduced with different doses of sdAb-encoding LVs ranging from 0.5 to 500 ng of RT in 700 μL of DMEM. Eighteen hours later, the transduction mixture was replaced with RPMI 1640. The cells were detached 48 h after transduction. A part of the cells was analyzed in flow cytometry to assess expression of PD-L1 and binding of sdAbs to PD-L1. A part of the cells was pulsed with 50 μg/mL gp100_280–288_ peptide, after which they were seeded in duplicate at 2 × 10^4^ cells per well of a 96-well round-bottom plate. 2D3 cells were electroporated with mRNA encoding the TCRα and TCRβ chain of a TCR recognizing the gp100_280–288_ peptide in the context of HLA-A2 (2.5 μg/10^6^ cells) with or without 2.5 μg of mRNA-encoding PD-1, as previously described.^20^ Expression of PD-1 and the TCR was confirmed by flow cytometry. A total of 2 × 10^5^ PD-1^POS^ or PD-1^NEG^, however, TCR^POS^ 2D3 cells were added per well to the 624-MEL cells. Expression of GFP by the 2D3 cells, which serves as a measure of TCR signaling, was determined by flow cytometry. The relative increase in TCR signaling was calculated as follows: (% GFP of PD-1^POS^ 2D3 cells/% GFP of PD-1^NEG^ 2D3 cells)_TRANSDUCED_ − (% GFP of PD-1^POS^ 2D3 cells/% GFP of PD-1^NEG^ 2D3 cells)_UNTRANSDUCED_.

### Flow Cytometry

Cell staining procedures were performed as described previously.^41^ Cells were acquired on the LSRFortessa flow cytometer (BD Biosciences). Data were analyzed using FACSDiva software (BD Biosciences) or FlowJo v10 (BD Biosciences).

### Production, purification, and quality control of sdAbs

#### In bacteria

sdAb production and purification were described earlier.^44^ Briefly, the DNA sequences of K2, 2K2, R3, and 2R3 were cloned *in silico* using SnapGene software. The sequences contained overhangs, allowing the ligation into the pHEN6 plasmid. DNA fragments were subsequently synthesized and produced by IDT. The pHEN6 vector was digested using PstI and BstEII, and the DNA fragments were ligated by Gibson assembly master mix according to the manufacturer’s instructions. The resulting plasmids were transformed into WK6 *E. coli* for large-scale production. sdAbs were purified from the periplasmatic extract as described earlier.^45^

#### In mammalian cells

293T cells were transduced using LVs encoding K2, 2K2, 3K2, 2K2-DS, or K2-Fc. Modified cells were expanded. Subsequently, the medium was replaced by Opti-MEM (Thermo Scientific). Supernatants were concentrated using Vivaspin columns (Sartorius, Germany), and SDS-PAGE was performed. After Coomassie blue staining gels were analyzed using the Odyssey Fc dual-mode imaging system (LI-COR Biosciences, USA), and quantities were estimated using band intensities and interpolation.

### SPR

The affinity of sdAbs was evaluated on immobilized human PD-L1 proteins, as described earlier.^16^ For the regeneration 0.5 M NaCl and 20 nM NaOH solution was used.

### Competition study

624-MEL PD-L1^POS^ cells were incubated with recombinant human PD-1-Fc protein (R&D Systems) or biotinylated recombinant human PD-1 (Abcam) and subsequently incubated with various amounts of PD-L1-binding sdAbs. Cells were stained for Fc or biotin and analyzed using flow cytometry.

### 3D melanoma killing assay

GFP^POS^ 624-MEL cells were cultured in ultra-low adherence plates (Greiner Bio-One and Costar) in 50 μL of DMEM (Sigma-Aldrich). After 48 h, LVs at an amount of 100 ng of RT were added in 50 μL of DMEM containing 20 μg/mL protamine sulfate. Another 48 h later, PBMCs were thawed and 3 × 10^5^ cells were added to the 3D tumors in RPMI 1640 containing 100 ng/mL IL-15 (PeproTech, USA). Green fluorescence confluence of 3D tumors was monitored every hour for 5 consecutive days using the IncuCyte Zoom live cell imaging system (EssenBio, UK). Each 3D tumor confluence was normalized to the 3D tumor confluence at 0 h. The percentage killing was calculated from the area under the curve (AUC) from 50 h onward as 1 – (AUC_TREATED_/AUC_MOCK_). For the evaluation of immune cell activation, a 3D melanoma killing assay was performed the same way, but with GFP^NEG^ 624-MEL cells. Immune cells were isolated once tumor size was visibly reduced, but not completely diminished. Cells were analyzed using flow cytometry.

### Statistical analysis

All statistical analyses were performed using GraphPad Prism software v8.4.3. p values were calculated using either a Student’s t test or a repeated measures one-way ANOVA with multiple comparison and a Fisher’s least significant difference (LSD) test. Statistical significance is indicated as follows: ∗p < 0.05, ∗∗p < 0.01, ∗∗∗p < 0.001, ∗∗∗∗p < 0.0001; n.s., not significant.

## Supplemental information

Document S1. Figures S1–S5

Document S2. Article plus supplemental information

## References

[1] Brahmer J.R., Drake C.G., Wollner I., Powderly J.D., Picus J., Sharfman W.H., Stankevich E., Pons A., Salay T.M., McMiller T.L. (2010). Phase I study of single-agent anti-programmed death-1 (MDX-1106) in refractory solid tumors: safety, clinical activity, pharmacodynamics, and immunologic correlates. J. Clin. Oncol..

[2] Topalian S.L., Hodi F.S., Brahmer J.R., Gettinger S.N., Smith D.C., McDermott D.F., Powderly J.D., Carvajal R.D., Sosman J.A., Atkins M.B. (2012). Safety, activity, and immune correlates of anti-PD-1 antibody in cancer. N. Engl. J. Med..

[3] Rimm D.L., Han G., Taube J.M., Yi E.S., Bridge J.A., Flieder D.B., Homer R., West W.W., Wu H., Roden A.C. (2017). A prospective, multi-institutional, pathologist-based assessment of 4 immunohistochemistry assays for PD-L1 expression in non–small cell lung cancer. JAMA Oncol..

[4] Duan J., Cui L., Zhao X., Bai H., Cai S., Wang G., Zhao Z., Zhao J., Chen S., Song J. (2020). Use of immunotherapy with programmed cell death 1 vs programmed cell death ligand 1 inhibitors in patients with cancer: A systematic review and meta-analysis. JAMA Oncol..

[5] McDermott D.F., Drake C.G., Sznol M., Choueiri T.K., Powderly J.D., Smith D.C., Brahmer J.R., Carvajal R.D., Hammers H.J., Puzanov I. (2015). Survival, durable response, and long-term safety in patients with previously treated advanced renal cell carcinoma receiving nivolumab. J. Clin. Oncol..

[6] Gettinger S.N., Horn L., Gandhi L., Spigel D.R., Antonia S.J., Rizvi N.A., Powderly J.D., Heist R.S., Carvajal R.D., Jackman D.M. (2015). Overall survival and long-term safety of nivolumab (anti-programmed death 1 antibody, BMS-936558, ONO-4538) in patients with previously treated advanced non-small-cell lung cancer. J. Clin. Oncol..

[7] Pen J.J., Keersmaecker B.D., Heirman C., Corthals J., Liechtenstein T., Escors D., Thielemans K., Breckpot K. (2014). Interference with PD-L1/PD-1 co-stimulation during antigen presentation enhances the multifunctionality of antigen-specific T cells. Gene Ther..

[8] Chen S., Crabill G.A., Pritchard T.S., McMiller T.L., Wei P., Pardoll D.M., Pan F., Topalian S.L. (2019). Mechanisms regulating PD-L1 expression on tumor and immune cells. J. Immunother. Cancer.

[9] Zhang X., Schwartz J.C.D., Guo X., Bhatia S., Cao E., Lorenz M., Cammer M., Chen L., Zhang Z.Y., Edidin M.A. (2004). Structural and functional analysis of the costimulatory receptor programmed death-1. Immunity.

[10] Arasanz H., Gato-Cañas M., Zuazo M., Ibañez-Vea M., Breckpot K., Kochan G., Escors D. (2017). PD1 signal transduction pathways in T cells. Oncotarget.

[11] Karwacz K., Bricogne C., MacDonald D., Arce F., Bennett C.L., Collins M., Escors D. (2011). PD-L1 co-stimulation contributes to ligand-induced T cell receptor down-modulation on CD8^+^ T cells. EMBO Mol. Med..

[12] Juneja V.R., McGuire K.A., Manguso R.T., LaFleur M.W., Collins N., Haining W.N., Freeman G.J., Sharpe A.H. (2017). PD-L1 on tumor cells is sufficient for immune evasion in immunogenic tumors and inhibits CD8 T cell cytotoxicity. J. Exp. Med..

[13] Lin H., Wei S., Hurt E.M., Green M.D., Zhao L., Vatan L., Szeliga W., Herbst R., Harms P.W., Fecher L.A. (2018). Host expression of PD-L1 determines efficacy of PD-L1 pathway blockade-mediated tumor regression. J. Clin. Invest..

[14] Diskin B., Adam S., Cassini M.F., Sanchez G., Liria M., Aykut B., Buttar C., Li E., Sundberg B., Salas R.D. (2020). PD-L1 engagement on T cells promotes self-tolerance and suppression of neighboring macrophages and effector T cells in cancer. Nat. Immunol..

[15] Gato-Cañas M., Zuazo M., Arasanz H., Ibañez-Vea M., Lorenzo L., Fernandez-Hinojal G., Vera R., Smerdou C., Martisova E., Arozarena I. (2017). PDL1 signals through conserved sequence motifs to overcome interferon-mediated cytotoxicity. Cell Rep..

[16] Broos K., Lecocq Q., Xavier C., Bridoux J., Nguyen T.T., Corthals J., Schoonooghe S., Lion E., Raes G., Keyaerts M. (2019). Evaluating a single domain antibody targeting human PD-L1 as a nuclear imaging and therapeutic agent. Cancers (Basel).

[17] Lecocq Q., De Vlaeminck Y., Hanssens H., D’Huyvetter M., Raes G., Goyvaerts C., Keyaerts M., Devoogdt N., Breckpot K. (2019). Theranostics in immuno-oncology using nanobody derivatives. Theranostics.

[18] Broos K., Lecocq Q., Keersmaecker B., Raes G., Corthals J., Lion E., Thielemans K., Devoogdt N., Keyaerts M., Breckpot K. (2019). Single domain antibody-mediated blockade of programmed death-ligand 1 on dendritic cells enhances CD8 T-cell activation and cytokine production. Vaccines (Basel).

[19] Lemaire M., D’Huyvetter M., Lahoutte T., Van Valckenborgh E., Menu E., De Bruyne E., Kronenberger P., Wernery U., Muyldermans S., Devoogdt N., Vanderkerken K. (2014). Imaging and radioimmunotherapy of multiple myeloma with anti-idiotypic nanobodies. Leukemia.

[20] Versteven M., Van den Bergh J.M.J., Broos K., Fujiki F., Campillo-Davo D., De Reu H., Morimoto S., Lecocq Q., Keyaerts M., Berneman Z. (2018). A versatile T cell-based assay to assess therapeutic antigen-specific PD-1-targeted approaches. Oncotarget.

[21] Massa S., Xavier C., De Vos J., Caveliers V., Lahoutte T., Muyldermans S., Devoogdt N. (2014). Site-specific labeling of cysteine-tagged camelid single-domain antibody-fragments for use in molecular imaging. Bioconjug. Chem..

[22] Zang B., Ren J., Li D., Huang C., Ma H., Peng Q., Ji F., Han L., Jia L. (2019). Freezing-assisted synthesis of covalent C-C linked bivalent and bispecific nanobodies. Org. Biomol. Chem..

[23] Shields R.L., Namenuk A.K., Hong K., Meng Y.G., Rae J., Briggs J., Xie D., Lai J., Stadlen A., Li B. (2001). High resolution mapping of the binding site on human IgG1 for FcγRI, FcγRII, FcγRIII, and FcRn and design of IgG1 variants with improved binding to the FcγR. J. Biol. Chem..

[24] June C.H., Warshauer J.T., Bluestone J.A. (2017). Is autoimmunity the Achilles’ heel of cancer immunotherapy?. Nat. Med..

[25] Naidoo J., Wang X., Woo K.M., Iyriboz T., Halpenny D., Cunningham J., Chaft J.E., Segal N.H., Callahan M.K., Lesokhin A.M. (2017). Pneumonitis in patients treated with anti-programmed death-1/programmed death ligand 1 therapy. J. Clin. Oncol..

[26] Ishihara J., Fukunaga K., Ishihara A., Larsson H.M., Potin L., Hosseinchi P., Galliverti G., Swartz M.A., Hubbell J.A. (2017). Matrix-binding checkpoint immunotherapies enhance antitumor efficacy and reduce adverse events. Sci. Transl. Med..

[27] Reul J., Frisch J., Engeland C.E., Thalheimer F.B., Hartmann J., Ungerechts G., Buchholz C.J. (2019). Tumor-specific delivery of immune checkpoint inhibitors by engineered AAV vectors. Front. Oncol..

[28] Mitchell L.A., Yagiz K., Hofacre A., Viaud S., Munday A.W., Espinoza F.L., Mendoza D., Rodriguez-Aguirre M.E., Bergqvist S., Haghighi A. (2019). PD-L1 checkpoint blockade delivered by retroviral replicating vector confers anti-tumor efficacy in murine tumor models. Oncotarget.

[29] Marabelle A., Andtbacka R., Harrington K., Melero I., Leidner R., de Baere T., Robert C., Ascierto P.A., Baurain J.F., Imperiale M. (2018). Starting the fight in the tumor: Expert recommendations for the development of human intratumoral immunotherapy (HIT-IT). Ann. Oncol..

[30] Lamichhane P., Deshmukh R., Brown J.A., Jakubski S., Parajuli P., Nolan T., Raja D., Badawy M., Yoon T., Zmiyiwsky M., Lamichhane N. (2019). Novel delivery systems for checkpoint inhibitors. Medicines (Basel).

[31] Emeagi P.U., Van Lint S., Goyvaerts C., Maenhout S., Cauwels A., McNeish I.A., Bos T., Heirman C., Thielemans K., Aerts J.L., Breckpot K. (2012). Proinflammatory characteristics of SMAC/DIABLO-induced cell death in antitumor therapy. Cancer Res..

[32] Milone M.C., O’Doherty U. (2018). Clinical use of lentiviral vectors. Leukemia.

[33] Sadeghnezhad G., Romão E., Bernedo-Navarro R., Massa S., Khajeh K., Muyldermans S., Hassania S. (2019). Identification of new DR5 agonistic nanobodies and generation of multivalent nanobody constructs for cancer treatment. Int. J. Mol. Sci..

[34] De Vlieger D., Ballegeer M., Rossey I., Schepens B., Saelens X. (2018). Single-domain antibodies and their formatting to combat viral infections. Antibodies (Basel).

[35] Chen X., Zaro J.L., Shen W.C. (2013). Fusion protein linkers: property, design and functionality. Adv. Drug Deliv. Rev..

[36] van Lith S.A.M., van Duijnhoven S.M.J., Navis A.C., Leenders W.P.J., Dolk E., Wennink J.W.H., van Nostrum C.F., van Hest J.C.M. (2017). Legomedicine-A versatile chemo-enzymatic approach for the preparation of targeted dual-labeled llama antibody-nanoparticle conjugates. Bioconjug. Chem..

[37] Tan S., Liu K., Chai Y., Zhang C.W.H., Gao S., Gao G.F., Qi J. (2018). Distinct PD-L1 binding characteristics of therapeutic monoclonal antibody durvalumab. Protein Cell.

[38] Zhang F., Wei H., Wang X., Bai Y., Wang P., Wu J., Jiang X., Wang Y., Cai H., Xu T., Zhou A. (2017). Structural basis of a novel PD-L1 nanobody for immune checkpoint blockade. Cell Discov..

[39] Gaiser M.R., Bongiorno M., Brownell I. (2018). PD-L1 inhibition with avelumab for metastatic Merkel cell carcinoma. Expert Rev. Clin. Pharmacol..

[40] Papadopoulos K.P., Harb W., Lu N., Ma X., He Y., Yuan L., Fu M., Lin Y., Xu W., Wang X. (2018). Phase I study of KN035, a novel fusion anti-PD-L1 antibody administered subcutaneously in patients with advanced solid tumors in the USA. Ann. Oncol..

[41] Breckpot K., Dullaers M., Bonehill A., van Meirvenne S., Heirman C., de Greef C., van der Bruggen P., Thielemans K. (2003). Lentivirally transduced dendritic cells as a tool for cancer immunotherapy. J. Gene Med..

[42] De Vlaeminck Y., Lecocq Q., Giron P., Heirman C., Geeraerts X., Bolli E., Movahedi K., Massa S., Schoonooghe S., Thielemans K. (2019). Single-domain antibody fusion proteins can target and shuttle functional proteins into macrophage mannose receptor expressing macrophages. J. Control. Release.

[43] Goyvaerts C., De Groeve K., Dingemans J., Van Lint S., Robays L., Heirman C., Reiser J., Zhang X.Y., Thielemans K., De Baetselier P. (2012). Development of the nanobody display technology to target lentiviral vectors to antigen-presenting cells. Gene Ther..

[44] Broisat A., Hernot S., Toczek J., De Vos J., Riou L.M., Martin S., Ahmadi M., Thielens N., Wernery U., Caveliers V. (2012). Nanobodies targeting mouse/human VCAM1 for the nuclear imaging of atherosclerotic lesions. Circ. Res..

[45] Lecocq Q., Zeven K., De Vlaeminck Y., Martens S., Massa S., Goyvaerts C., Raes G., Keyaerts M., Breckpot K., Devoogdt N. (2019). Noninvasive imaging of the immune checkpoint LAG-3 using nanobodies, from development to pre-clinical use. Biomolecules.

