# Supplementary material for: Formatting and gene-based delivery of a human PD-L1 single domain antibody for immune checkpoint blockade
Source: Mol Ther Methods Clin Dev. 2021 Jun 4;22:172–82. doi: 10.1016/j.omtm.2021.05.017 (PMC8397838; doi:10.1016/j.omtm.2021.05.017)
Supplement: Document S1. Figures S1–S5 [file mmc1.pdf]

OMTM, Volume *22*

Supplemental information

Formatting and gene-based delivery of a human PD-L1 single domain antibody for immune checkpoint blockade

Robin Maximilian Awad, Quentin Lecocq, Katty Zeven, Thomas Ertveldt, Lien De Beck, Hannelore Ceuppens, Katrijn Broos, Yannick De Vlaeminck, Cleo Goyvaerts, Magali Verdonck, Geert Raes, Alexander Van Parys, Anje Cauwels, Marleen Keyaerts, Nick Devoogdt, and Karine Breckpot

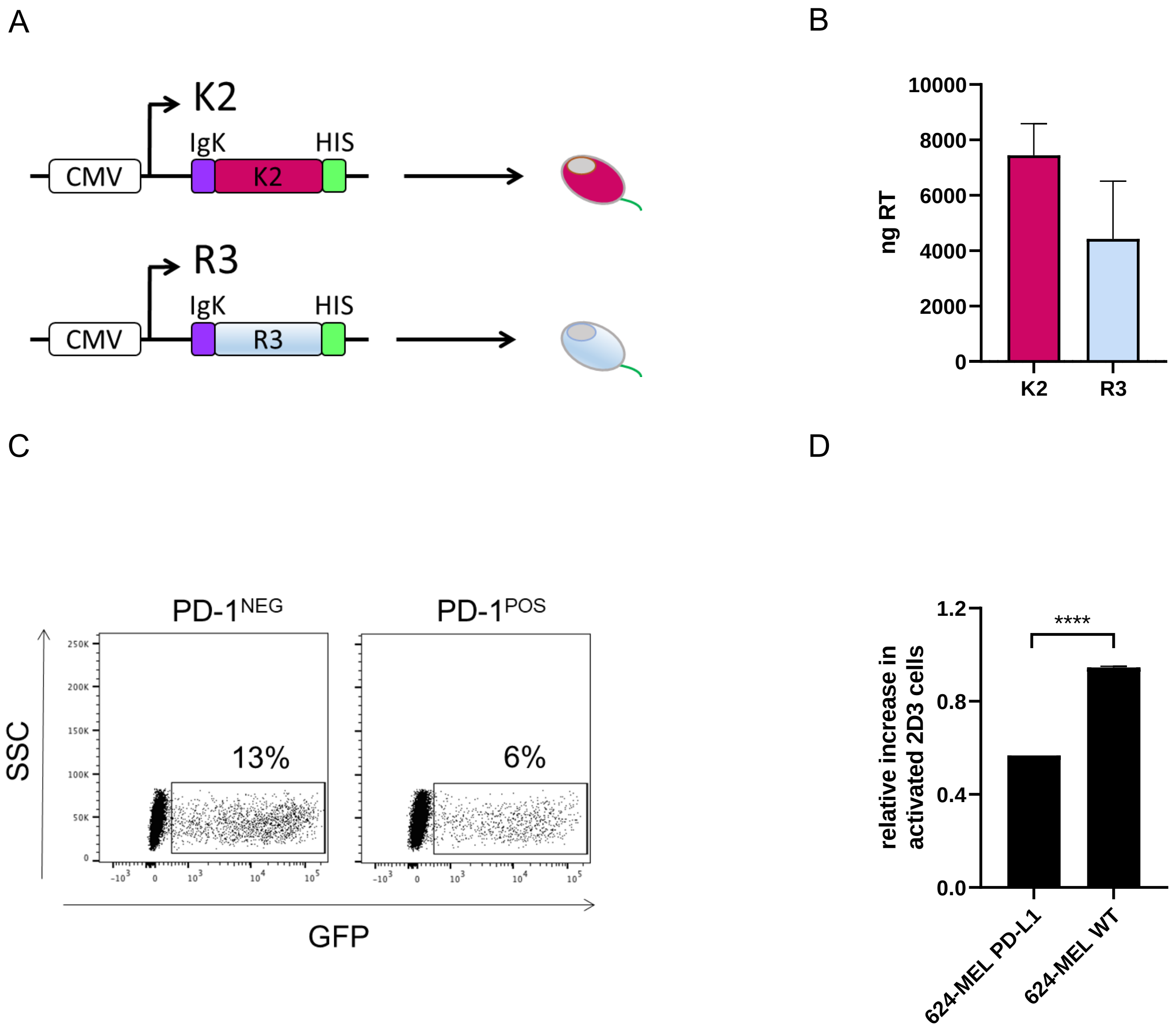

Figure S1. Generation of K2 (anti-PD-L1) and R3 (control sdAb)-encoding LVs. (A) Schematically annotated representation of sdAbs K2 and R3 and schematic representation of secreted sdAbs K2 and R3, CMV; Cytomegalovirus promoter, Igκ; murine Igκ leader sequence, HIS; 6x Histidine tag. (B) 293T cells were transfected in order to produce LVs. LVs were concentrated and colorimetric reverse transcriptase assay was performed. The graph depicts the amount reverse transcriptase (ng RT) in the LV productions. (C) 2D3 PD-1^{NEG} and 2D3 PD-1^{POS} cells were co-cultured with PD-L1^{POS} 624-MEL cells. GFP expression was evaluated by flow cytometry. (D) 2D3 PD-1^{NEG} and 2D3 PD-1^{POS} cells were co-cultured with 624-MEL PD-L1^{NEG} and 624-MEL PD-L1^{POS} cells. GFP expression was evaluated by flow cytometry (n=2).

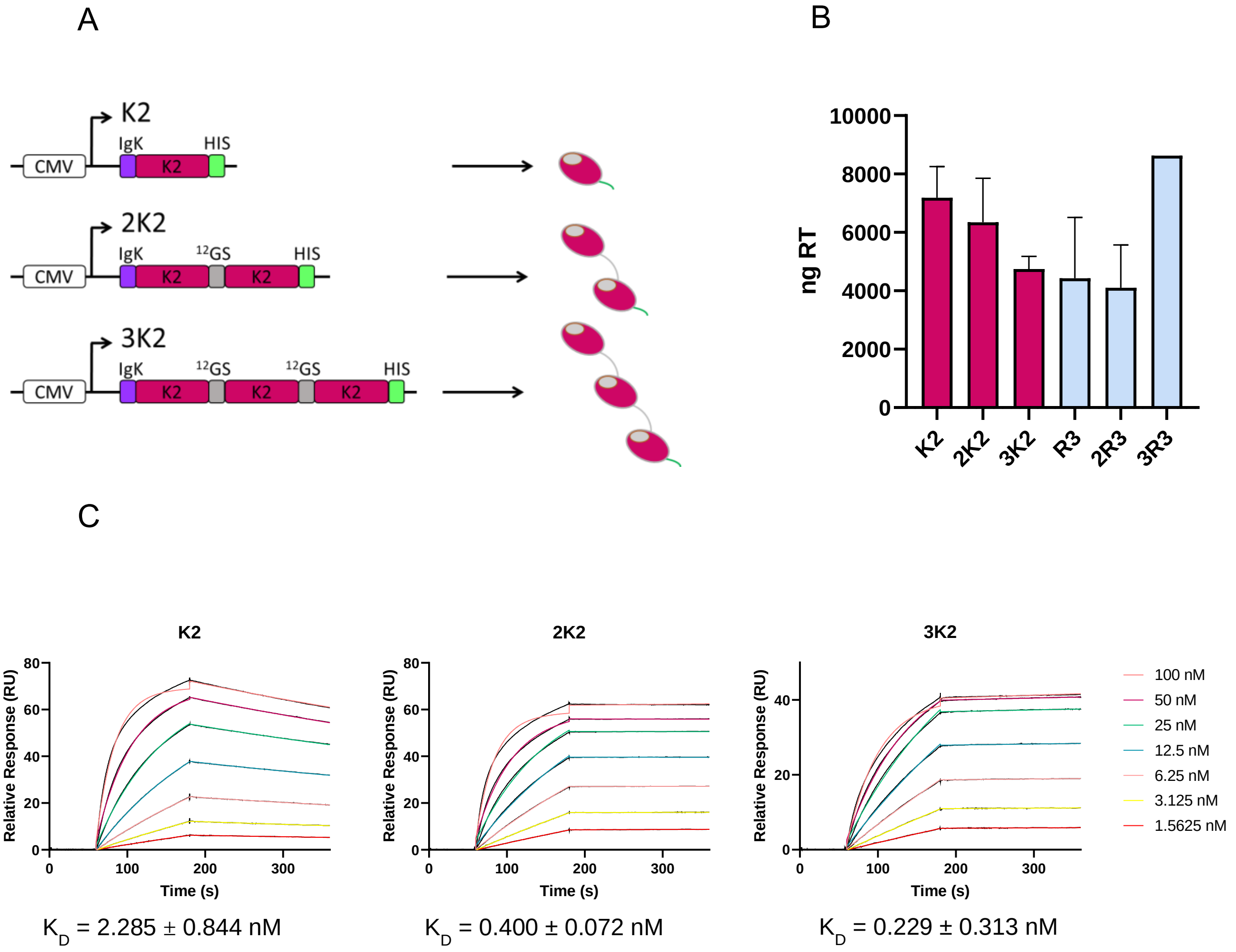

Figure S2. Generation of K2, 2K2 and 3K2 encoding LVs and evaluation of binding kinetics. (A) Schematically annotated representation of sdAb formats K2, 2K2 and 3K2 and schematic representation of secreted sdAb formats K2, 2K2 and 3K2, CMV; Cytomegalovirus promoter, Igκ; murine Igκ leader sequence, 12GS: 12 Glycine-Serine linker, HIS; 6x Histidine tag. (B) 293T cells were transfected in order to produce K2, 2K2 or 3K2 encoding LVs. LVs were concentrated and colorimetric reverse transcriptase assay was performed. The graph depicts the amount reverse transcriptase (ng RT) in the LV productions. (C) 293T cells were transduced with LVs encoding K2, 2K2 and 3K2. Subsequently, sdAb-containing supernatants were collected, concentrated, and quantified. The affinity/kinetics of sdAbs on immobilized recombinant human PD-L1 protein were analyzed. One representative graph is shown together with the K_D ± SD (n=2).

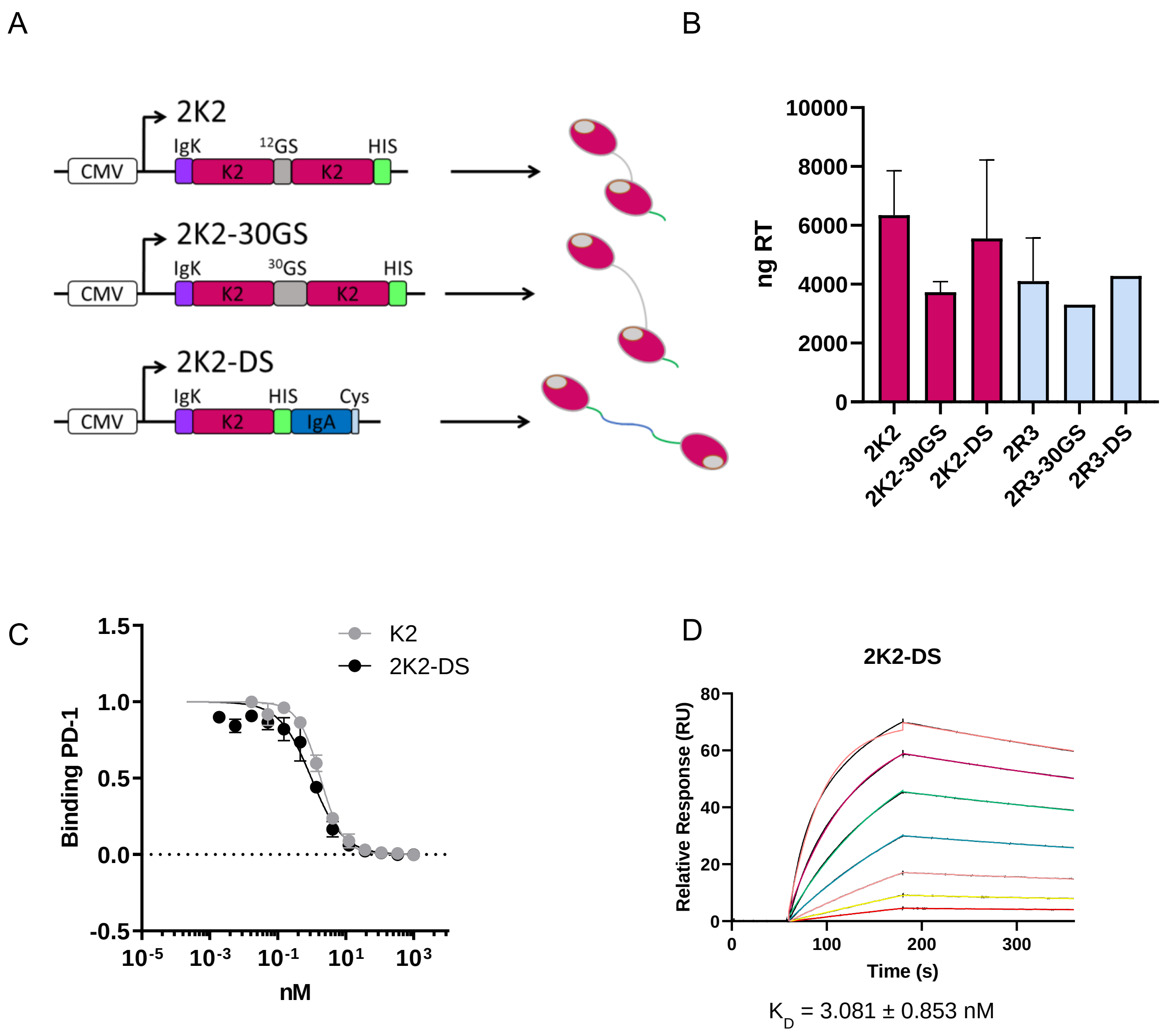

Figure S3. Generation of 2K2, 2K2-30GS and 2K2-DS encoding LVs and evaluation of binding kinetics. (A) Schematically annotated representation of sdAb formats 2K2, 2K2-30GS and 2K2-DS, CMV; Cytomegalovirus promoter, Igκ; murine Igκ leader sequence, 12GS; 12 Glycine-Serine linker, 30GS; 30 Glycerine-Serine linker, IgA; IgA-hinge linker, Cys; Cysteine residue, 6xHis; 6x Histidine tag and schematic representation of secreted sdAb formats 2K2, 2K2-30GS and 2K2-DS. (B) 293T cells were transfected in order to produce 2K2, 2K2-30GS or 2K2-DS encoding LVs. LVs were concentrated and colorimetric reverse transcriptase assay was performed. The graph depicts the amount reverse transcriptase (ng RT) in the LV productions. (C) 293T cells were transduced with LVs encoding 2K2-DS. Subsequently, sdAb-containing supernatants were collected, concentrated, and quantified. 624-MEL PD-L1^{POS} cells were incubated with recombinant human PD-1-Fc protein and subsequently exposed to increasing amounts of 2K2-DS (n=3). The MFI of anti-Fc antibodies was measured using flow cytometry. (D) The affinity/kinetics of 2K2-DS on immobilized recombinant human PD-L1 protein was analyzed using SPR. One representative graph is shown together with the K_D ± SD (n=2).

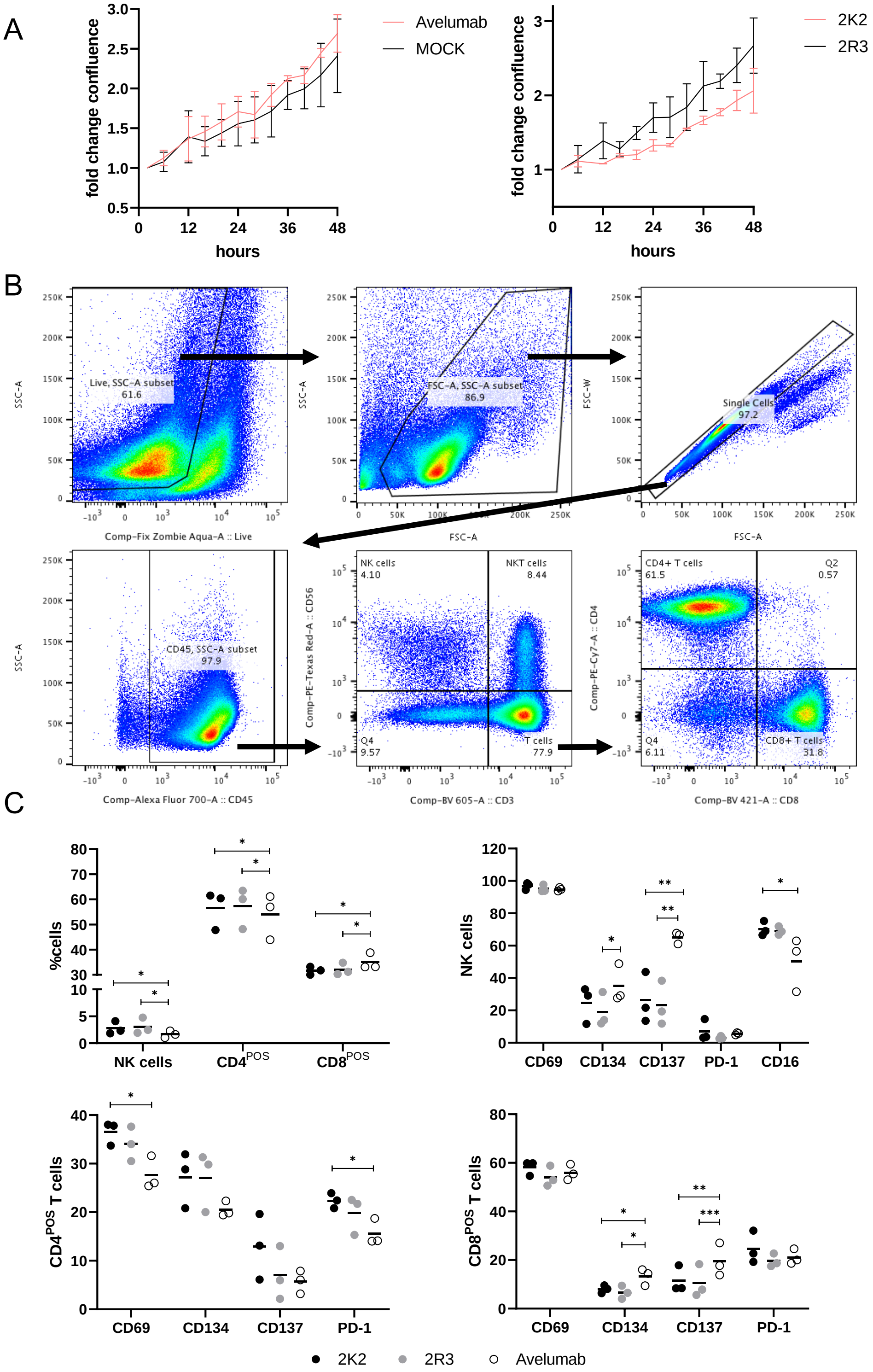

Figure S4. Immune cell activation in presence of 2K2 and Avelumab in co-culture with tumor cells. (A) 624-MEL PD-L1^{POS} cells were cultured in presence of 2K2, 2R3 or avelumab. Confluence was followed up using the IncuCyte Zoom live cell imaging system (n=2). (B) Gating strategy for discrimination between NK, CD4^{POS} and CD8^{POS} T cells. (C) Activated PBMCs were added to 624-MEL cells that were grown in 3D tumors. 3D tumors were transduced with LVs encoding 2K2 or 2R3 or were treated with avelumab. Once spheroid size was visible reduced, immune cells were isolated and analyzed using flow cytometry (n=3). The number of asterisks in the figure indicates the statistical significance as follows: * p < 0.05; ** p < 0,01; *** p < 0.001.

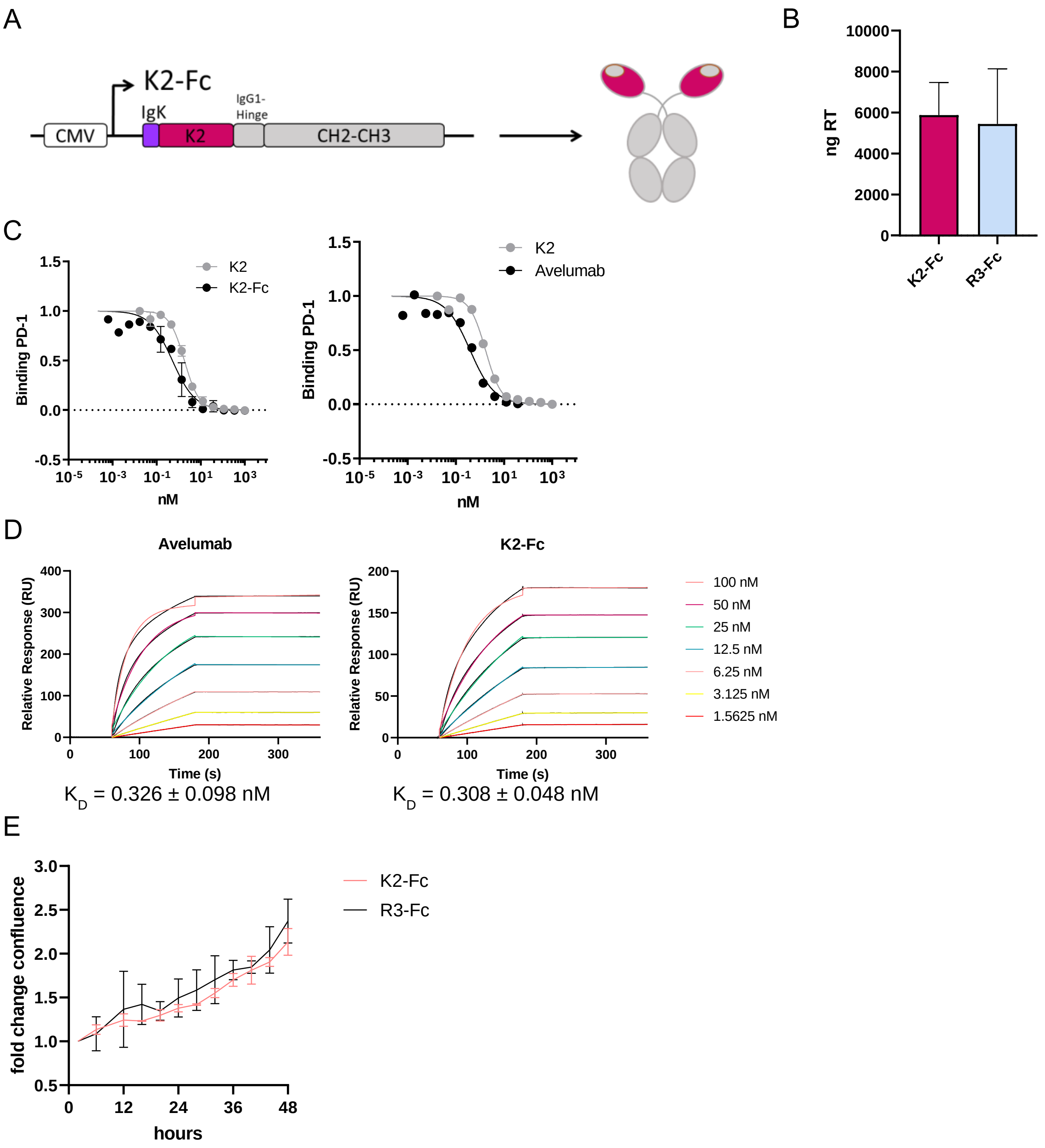

Figure S5. Generation of K2-Fc encoding LVs. (A) Schematically annotated representation of K2-Fc and schematic representation of secreted K2-Fc, CMV; Cytomegalovirus promoter, Igκ; murine Igκ leader sequence, CH2-CH3; human IgG1 Fc-tail. (B) 293T cells were transfected in order to produce K2-Fc or R3-Fc encoding LVs. LVs were concentrated and colorimetric reverse transcriptase assay was performed. The graph depicts the amount reverse transcriptase (ng RT) in the LV productions. (C) 293T cells were transduced with LVs encoding K2-Fc. Subsequently, sdAb-containing supernatants were collected, concentrated, and quantified. 624-MEL PD-L1^{POS} cells were incubated with recombinant biotinylated human PD-1 protein and subsequently exposed to increasing amounts of K2-Fc or avelumab (n=1-2). The MFI of PE Streptavidin was measured using flow cytometry. (D) The affinity/kinetics of K2-Fc on immobilized recombinant human PD-L1 protein was analyzed using SPR. One representative graph is shown together with the K_D ± SD (n=2). (E) 624-MEL PD-L1^{POS} cells were cultured in presence of K2-Fc or R3-Fc. Confluence was followed up using the IncuCyte Zoom live cell imaging system (n=2).
